# Efficacy of adjunctive oral Janus kinase inhibitors in hospitalized patients with extensive or difficult-to-control drug eruptions: a retrospective observational case series

**DOI:** 10.3389/fmed.2026.1947886

**Published:** 2026-10-26

**Authors:** Yunkai Zhu, Xia Wang, Yuhang Huang, Ming Wang, Lixian Lin, Jinsheng Li, Peiyi Feng, Yuan Lu, Xiaoming Liu, Bao Chai, Peng Wang

**Affiliations:** Department of Dermatology, Shenzhen Nanshan People’s Hospital/Affiliated Nanshan Hospital of Shenzhen University, Shenzhen, Guangdong, China

**Keywords:** body surface area, corticosteroid reduction, DRESS, drug eruption, janus kinase inhibitor, pruritus, rescue treatment

## Abstract

**Background:**

Evidence for Janus kinase (JAK) inhibition in severe or difficult-to-control drug eruptions remains limited, particularly regarding early response kinetics and corticosteroid reduction.

**Methods:**

This single-center retrospective observational case series included nine hospitalized patients with extensive or difficult-to-control drug eruptions treated with adjunctive oral JAK inhibitors between March 2023 and January 2026. Cases 1–7 formed a rescue treatment subgroup because active progression persisted after at least 48 h of systemic corticosteroids. The primary outcome was the first documented absence of new lesions within 72 h. Secondary outcomes included serial changes in Worst Itch Numerical Rating Scale (WI-NRS) and body surface area (BSA), time to corticosteroid reduction, recurrence, and safety. Within-patient changes were assessed using Friedman tests, with exact Wilcoxon signed-rank tests and Holm correction for *post hoc* comparisons.

**Results:**

The rescue subgroup received systemic corticosteroids for a median of 72 h before JAK inhibitor initiation (IQR, 48–96 h). New lesions were first documented as absent within 24 h in 6/7 patients and within 48 h in all seven. All seven began corticosteroid reduction within 96 h (median, 48 h). Across all nine patients, median WI-NRS decreased from 6 at baseline to 1 at 72 h [Friedman *χ*^2^(4) = 35.04, *P* < 0.001; Kendall's *W* = 0.973], and all patients achieved a reduction of at least 3 points. Median BSA decreased from 55% at baseline to 30% at day 3 and 10% by day 7 or discharge [Friedman *χ*^2^(2) = 18.00, *P* < 0.001; Kendall's *W* = 1.000]. Two herpes simplex-related events occurred during combined immunosuppression.

**Conclusion:**

Adjunctive oral JAK inhibitors were associated with rapid control of new-lesion formation, substantial improvement in pruritus and cutaneous involvement, and early corticosteroid reduction. These findings support JAK inhibition as a promising rescue or steroid-sparing option for selected difficult-to-control drug eruptions and warrant prospective controlled evaluation.

## 1 Introduction

Cutaneous adverse drug reactions encompass a broad clinical spectrum, ranging from self-limited maculopapular eruptions to severe cutaneous adverse reactions, including drug reaction with eosinophilia and systemic symptoms (DRESS), acute generalized exanthematous pustulosis (AGEP), and Stevens–Johnson syndrome/toxic epidermal necrolysis (SJS/TEN) (1–4). Early presentations may be non-specific, and an apparently uncomplicated exanthem may continue to evolve before visceral injury, pustulation, mucosal involvement, or epidermal detachment becomes apparent. Prompt withdrawal of the suspected culprit medication and supportive care remain fundamental. However, extensive or rapidly progressive involvement, severe pruritus, facial edema, fever, hematologic abnormalities, or internal-organ involvement may require systemic treatment (1–5).

Systemic corticosteroids are widely used for severe or progressive drug eruptions, particularly DRESS, but the supporting evidence is predominantly observational (2, 4, 5). Clinical response may be incomplete or delayed, and prolonged treatment can be complicated by relapse, infection, hyperglycemia, hypertension, and other corticosteroid-related toxicities (2, 4, 5). Etanercept has demonstrated benefit in a randomized trial of SJS/TEN, but its role in DRESS, AGEP, and extensive maculopapular eruptions remains uncertain (6). Consequently, the optimal management of patients who continue to develop new lesions or show increasing erythema, edema, cutaneous extent, or pruritus despite culprit drug withdrawal and systemic corticosteroid treatment remains poorly defined. A rapidly acting adjunctive treatment that enables earlier disease control and subsequent corticosteroid reduction would therefore address an important clinical need.

The Janus kinase-signal transducer and activator of transcription (JAK-STAT) pathway represents a biologically plausible therapeutic target in delayed drug hypersensitivity. Drug-responsive T cells generate overlapping type 1, type 2, cytotoxic, and pruritic cytokine programs, many of which depend on JAK-STAT signaling. Single-cell transcriptomic analysis demonstrated pathway activation in corticosteroid-refractory drug-induced hypersensitivity syndrome/DRESS and guided successful treatment with tofacitinib (7). Recalcitrant DRESS has also been associated with CCR8/CCL1-mediated type 2 memory responses and CXCR3/CXCL10-mediated cytotoxic memory T-cell activation, both of which were attenuated by JAK inhibition in functional experiments (8). Spatial proteomic analysis of TEN further identified interferon-STAT1 signaling as a major pathogenic program (9). These findings suggest that JAK inhibition may suppress several downstream inflammatory circuits simultaneously rather than targeting an individual cytokine.

Clinical evidence nevertheless remains limited. Tofacitinib has been reported in severe DRESS with myocardial involvement (10), and a six-patient series of recurrent or refractory DRESS treated with baricitinib or abrocitinib described rapid improvement in cutaneous lesions and pruritus and initiation of corticosteroid tapering within 7–12 days (11). JAK inhibition has also been used in persistent erythema multiforme associated with interferon-gamma- and interleukin-15-related inflammation (12). However, most published evidence consists of individual cases or small series focused on a single phenotype, and little is known about treatment sequence, early response kinetics, or corticosteroid reduction patterns when oral JAK inhibitors are added to ongoing treatment for extensive or difficult-to-control drug eruptions.

We therefore conducted a single-center retrospective observational case series of nine hospitalized patients with extensive or difficult-to-control drug eruptions treated with adjunctive oral JAK inhibitors. We reconstructed treatments administered before JAK inhibitor initiation, distinguished a rescue treatment subgroup from other clinically informative treatment contexts, and assessed early cessation of new-lesion formation, serial changes in pruritus and BSA involvement, time to systemic corticosteroid reduction, recurrence, and safety.

## 2 Materials and methods

### 2.1 Study design and participants

This retrospective observational case series was conducted at the Department of Dermatology, Shenzhen Nanshan People's Hospital/Affiliated Nanshan Hospital of Shenzhen University. We reviewed all hospitalized patients with a clinically diagnosed drug eruption who received an oral JAK inhibitor between March 2023 and January 2026.

Patients were eligible when they fulfilled all of the following criteria: (1) a plausible recent medication exposure; (2) a clinical morphology and exposure-to-onset chronology compatible with a drug eruption; (3) documented administration of an oral JAK inhibitor during hospitalization; and (4) sufficient contemporaneous records to reconstruct the clinical phenotype, treatment sequence, quantitative early assessments, and outcome. Patients were excluded when an alternative diagnosis better explained the eruption or when the available documentation was insufficient for clinical evaluation. All nine eligible hospitalized patients identified during the study period were included.

### 2.2 Diagnostic assessment and data collection

Clinical phenotypes were assigned according to suspected medication exposure, exposure-to-onset latency, lesion morphology, BSA involvement, fever, facial edema, lymphadenopathy, mucosal or epidermal involvement, hematologic abnormalities, and hepatic or renal involvement. The RegiSCAR scoring system was applied to suspected DRESS. Cases 8 and 9 were classified as definite DRESS. The EuroSCAR framework was considered when classifying the probable AGEP in Case 6.

Case 4 was classified as a suspected maculopapular eruption superimposed on erythrodermic atopic dermatitis because preexisting erythroderma, concurrent infection, and exposure to multiple treatments substantially confounded diagnostic interpretation. Its baseline BSA value was therefore considered to represent mixed inflammatory skin burden rather than the suspected drug eruption alone.

Data were extracted from electronic medical records, medication administration records, laboratory reports, progress notes, follow-up records, and contemporaneous clinical photographs. Two dermatologists independently verified the clinical phenotype, suspected culprit medication, treatment timeline, WI-NRS and BSA assessments, new-lesion status, corticosteroid dose changes, recurrence, and safety outcomes. Disagreements were resolved by consensus.

### 2.3 Treatment exposure and treatment context classification

Day 0 was defined as the date of the first actual administration of an oral JAK inhibitor. Pre-JAK systemic corticosteroid exposure was calculated from the first documented administration of systemic corticosteroids to JAK inhibitor initiation. Etanercept and intravenous immunoglobulin (IVIG) were classified according to whether they had been administered before JAK initiation or during the same treatment escalation phase.

All patients received methylprednisolone as the systemic corticosteroid at JAK inhibitor initiation. To facilitate comparison across patients, the dose at JAK inhibitor initiation was additionally expressed in mg/kg/day using the recorded admission weight from the index hospitalization.

Suspected culprit medications were withdrawn whenever clinically feasible. Background treatment was individualized and included antihistamines, topical or systemic corticosteroids, sodium thiosulfate, etanercept, IVIG, antiviral or antibacterial therapy, and supportive care. Oral JAK inhibitors were prescribed off label as treatment escalation. The selected agent, initial dose, subsequent dose adjustment, switching, treatment duration, and discontinuation were determined clinically according to phenotype, disease severity, comorbidities, treatment response, safety considerations, affordability, and the anticipated need for continued disease control. In practice, affordability and clinical experience were major considerations, with tofacitinib and baricitinib offering lower-cost options in our setting. Upadacitinib or abrocitinib were selected in some patients when affordable, based on their JAK1 preferential profiles and the anticipated benefit of targeting JAK1-dependent inflammation. Published experience with JAK inhibition in drug eruptions also informed these choices (7, 10). No standardized JAK inhibitor protocol or phenotype-specific selection algorithm was used.

Patients were retrospectively categorized according to the clinical context in which JAK inhibition was initiated. The rescue treatment subgroup comprised patients who had received systemic corticosteroids for at least 48 h and had documented active progression during the 24 h immediately preceding JAK inhibitor initiation. Active progression required continued new-lesion formation together with at least one additional feature: expanding BSA involvement, worsening erythema or edema, or uncontrolled severe pruritus. Cases 1–7 fulfilled these criteria.

Case 8 was categorized as residual high disease burden with steroid-sparing escalation because new-lesion formation had ceased before JAK initiation, but extensive erythema, desquamation, pruritus, and substantial corticosteroid exposure persisted. Case 9 was categorized as early multidrug co-escalation because baricitinib, systemic corticosteroids, and IVIG were initiated during the same treatment escalation phase for severe multisystem DRESS. This sequence did not permit classification as rescue treatment after an inadequate response to corticosteroids. These categories were assigned retrospectively to describe treatment sequence and were not prespecified or validated clinical classifications.

### 2.4 Outcome assessment

The primary outcome was the first documented absence of new-lesion formation within 72 h after JAK inhibitor initiation in the rescue treatment subgroup. New-lesion status was assessed from routine clinical documentation at approximately 24, 48, and 72 h after Day 0 and was categorized as present, absent, or undocumented.

Time to the first documented absence of new lesions was assigned only when new-lesion formation was present at or immediately before JAK inhibitor initiation. Because the assessments were retrospective and intermittent rather than continuous, this endpoint represented the first routine assessment documenting no further lesion formation and not the exact biological time at which lesion formation ceased.

Secondary outcomes included changes in WI-NRS, changes in BSA involvement, time to the first reduction in systemic corticosteroid dose, clinical recurrence, and safety. WI-NRS was assessed on a scale from 0 to 10 at baseline and approximately 24, 48, and 72 h after JAK initiation and at day 7 or discharge. A reduction of at least 3 points by 72 h was used as a descriptive threshold for clinically important improvement. BSA involvement was assessed at baseline, day 3, and day 7 or discharge. Day 7/discharge represented the day-7 assessment or the final inpatient assessment before discharge, whichever occurred first.

For patients receiving systemic corticosteroids, time to the first dose reduction was calculated from JAK inhibitor initiation to the first documented decrease in the total daily systemic corticosteroid dose. A reduction occurring on the same calendar day as JAK initiation would have been recorded as 0 h.

The primary new-lesion analysis was restricted to Cases 1–7. Case 8 was excluded because new-lesion formation had already ceased before JAK initiation. Case 9 was excluded because systemic corticosteroids, IVIG, and baricitinib were initiated concurrently. Cases 8 and 9 were retained in the overall analyses of WI-NRS and BSA because serial quantitative assessments were available.

### 2.5 Safety assessment

Safety outcomes included clinically apparent infection, herpesvirus-related events, thrombosis, major cardiovascular events, hyperglycemia, hematologic abnormalities, and hepatic or renal deterioration during treatment and available follow-up. An event was attributed to JAK inhibition only when the clinical record supported a plausible treatment relationship. Missing laboratory or follow-up data were recorded as unavailable and were not interpreted as absence of an adverse event.

### 2.6 Statistical analysis

Continuous variables are presented as medians with interquartile ranges (IQRs) and ranges, and categorical variables as numbers and percentages with explicit denominators. Exact 95% confidence intervals (CIs) for key proportions were calculated using the Clopper–Pearson method.

Exploratory within-patient changes in WI-NRS across baseline, 24 h, 48 h, 72 h, and day 7 or discharge were assessed using the Friedman test. Changes in BSA involvement across baseline, day 3, and day 7 or discharge were assessed using the same method. When the overall Friedman test was significant, baseline was compared with each follow-up assessment using two-sided Wilcoxon signed-rank tests. Exact *P* values were calculated by exhaustive sign permutations of the paired differences, with Holm correction applied separately within each outcome. Kendall's *W* was calculated as a measure of effect size.

Because the baseline BSA in Case 4 predominantly reflected preexisting erythrodermic atopic dermatitis, a sensitivity analysis excluding this patient was performed. The analysis of time to the first documented absence of new lesions was restricted to the rescue treatment subgroup. New-lesion outcomes and time to corticosteroid reduction were summarized descriptively because no untreated or conventional treatment comparator group was available. Overall time effects were additionally checked at revision using 100,000 within-patient permutations of time point labels.

Missing values were not imputed, and denominators were reported for each assessment. All tests were two-sided, with *P* < 0.05 considered statistically significant. Given the small sample size, retrospective outcome reconstruction, concomitant therapies, and uncontrolled design, the statistical analyses were interpreted as evidence of within-patient temporal change rather than evidence of an independent JAK inhibitor treatment effect. The revised analyses were performed using Python version 3.13.5, SciPy version 1.17.0, and statsmodels version 0.14.6.

### 2.7 Ethics

The study was approved by the Ethics Committee of Shenzhen Nanshan People's Hospital on June 30, 2026 (approval no. ky-2026-063001) and was conducted in accordance with the Declaration of Helsinki. The requirement for informed consent to participate was waived because of the retrospective study design. Written informed consent for publication of clinical photographs was obtained from Cases 2, 8, and 9.

## 3 Results

### 3.1 Patient characteristics and treatment contexts

Nine hospitalized patients were included, comprising five men and four women with a median age of 52 years (range, 43–78 years). Clinical phenotypes included extensive maculopapular or morbilliform eruptions with or without DRESS-like features, two cases of definite DRESS, one erythema multiforme-like drug eruption, one probable AGEP, and one suspected maculopapular eruption superimposed on erythrodermic atopic dermatitis.

Baseline BSA involvement ranged from 30% to 90%. The 90% value in Case 4 predominantly reflected preexisting erythrodermic atopic dermatitis and was addressed in a sensitivity analysis.

Four oral JAK inhibitors were used: upadacitinib in three patients, tofacitinib in three, abrocitinib in two, and baricitinib in one. Cases 1–7 formed the rescue treatment subgroup. Case 8 represented residual high disease burden with steroid-sparing escalation and Case 9 represented early multidrug co-escalation in severe DRESS. At JAK inhibitor initiation, methylprednisolone doses ranged from 0.67 to 1.90 mg/kg/day across the nine patients. Baseline characteristics and treatment contexts are summarized in Table 1, and patient-level treatment sequences and outcomes are shown in Table 2.

**Table 1:** Baseline characteristics and treatment contexts of the nine hospitalized patients.

Case	Age/sex	Suspected culprit drug(s); latency	Clinical phenotype	Baseline BSA (%)	Key clinical and laboratory features	Treatment context at JAK initiation
1	51/M	Unidentified NSAID; 7 d	Severe MPE with DRESS-like features (possible DRESS)	55	Facial and auricular edema; eosinophils 0.58 × 10^9/L; atypical lymphocytes 5%; mild isolated GGT elevation; no definite organ involvement	Rescue treatment subgroup
2	52/F	Tripterygium glycosides, more likely, and thalidomide; 7 and 12 days	Severe MPE with DRESS-like features	60	Facial and scalp edema with desquamation; eosinophils 0.94 × 10^9/L; atypical lymphocytes 9%; hypoalbuminemia; no definite hepatic or renal involvement	Rescue treatment subgroup
3	43/F	Amoxicillin and clarithromycin; approximately 1 day	Severe MPE	55	No mucosal or epidermal involvement; neutrophilic leukocytosis; atypical lymphocytes 1%; no eosinophilia or hepatic/renal dysfunction	Rescue treatment subgroup
4	50/M	Ceftriaxone, most suspected; IVIG also possible; approximately 2 and 1 day	Suspected MPE superimposed on erythrodermic AD	90^a^	Progressive diffuse erythema; background HSV-2 eczema herpeticum and bacterial skin infection; eosinophils peaked at 2.62 × 10^9/L; transient transaminitis; no renal deterioration	Rescue treatment subgroup
5	59/M	Amoxicillin, most likely, plus multiple concomitant drugs; approximately 1 day	Severe morbilliform/MPE	30	Ankle and wrist edema; no eosinophilia or hepatic dysfunction; stable preexisting chronic renal impairment	Rescue treatment subgroup
6	71/F	Etoricoxib; approximately 1 day	Probable AGEP	50	Fever to 38.5 °C; facial edema; diffuse pinpoint non-follicular pustules and desquamation; WBC 18.4 × 10^9/L; neutrophils 14.76 × 10^9/L; CRP 44.36 mg/L; no definite organ involvement	Rescue treatment subgroup
7	52/M	Methimazole, propranolol, or leucogen; undetermined; 10 days	Severe EM-like drug eruption	40	Atypical targetoid lesions with mild tenderness; no palmoplantar or mucosal involvement; no eosinophilia or definite organ involvement; infectious triggers not comprehensively tested	Rescue treatment subgroup
8	64/M	Carbamazepine, most suspected; pregabalin and vitamin B1 also possible; exposure within preceding 2 months	Definite DRESS, RegiSCAR score 7, without definite internal-organ involvement	60	Fever to 38.9 °C; facial and eyelid edema; generalized lymphadenopathy; eosinophils peaked at 2.24 × 10^9^/L; atypical lymphocytes 1%; no definite hepatic or renal involvement	Residual high disease burden with steroid-sparing escalation
9	78/F	Voriconazole; 28 days	Definite DRESS, RegiSCAR score 7, with hepatic and renal involvement	60	Fever to 38.5 °C; eyelid edema; axillary and supraclavicular lymphadenopathy; eosinophils peaked at 2.81 × 10^9^/L; atypical lymphocytes 2%; marked mixed/cholestatic liver injury and acute renal deterioration	Early multidrug co-escalation

AD, atopic dermatitis; AGEP, acute generalized exanthematous pustulosis; BSA, body surface area; CRP, C-reactive protein; DRESS, drug reaction with eosinophilia and systemic symptoms; EM, erythema multiforme; GGT, gamma-glutamyl transferase; HSV, herpes simplex virus; IVIG, intravenous immunoglobulin; JAK, Janus kinase; MPE, maculopapular eruption; NSAID, non-steroidal anti-inflammatory drug; WBC, white blood cell count.

a The 90% BSA value in Case 4 predominantly reflected preexisting erythrodermic AD rather than the suspected superimposed drug eruption alone. In Case 8, exact exposure dates were unavailable.

**Table 2:** JAK-containing treatment sequence and patient-level clinical outcomes.

Case	Initial JAK regimen	Pre-JAK treatment and MP dose at JAK initiation	WI-NRS: baseline → 72 h → day 7/discharge	BSA: baseline → day 3 → day 7/discharge	First documented absence of new lesions	First corticosteroid reduction	JAK course and notable outcome or safety event
1	Abrocitinib 200 mg qd	Corticosteroids 96 h; MP 60 mg/day (0.81 mg/kg/day); etanercept before JAK	5 → 1 → 0	55% → 20% → 5%	24 h	48 h; 60→40 mg/day	11 days, then stopped; no recurrence at 2 weeks; no clinical AE; post-discharge laboratory testing unavailable
2	Abrocitinib 200 mg qd	Corticosteroids 72 h; MP 80 mg/day (1.90 mg/kg/day); no etanercept or IVIG	8 → 3 → 1	60% → 30% → 10%	24 h	24 h; 80→40 mg/day	Abrocitinib 6 days, then changed to tofacitinib because of cost and continued mainly for dermatomyositis; no recurrence or clinical AE documented
3	Tofacitinib 5 mg bid; temporarily increased to 10 mg tid, then 5 mg tid	Corticosteroids 48 h; MP 80 mg/day (1.50 mg/kg/day); etanercept at JAK initiation	7 → 4 → 2	55% → 30% → 15%	24 h	72 h	6 days; stopped at discharge; no recurrence at 1 wk; no reported AE
4	Upadacitinib 15 mg qd	Corticosteroids 48 h; MP 40 mg/day (0.91 mg/kg/day); IVIG before JAK	7 → 2 → 1	90%* → 50% → 30%	24 h	24 h; 40→30 mg/day	15 days, then stopped; no recurrence by May 8; recurrent HSV-2 during combined immunosuppression
5	Tofacitinib 5 mg bid	Corticosteroids 96 h; MP 60 mg/day (0.86 mg/kg/day); etanercept before JAK	5 → 1 → 1	30% → 10% → 5%	24 h	24 h; 60→40 mg/day	9 days; stopped during corticosteroid taper; no recurrence or clinical AE documented
6	Upadacitinib 15 mg qd	Corticosteroids 48 h; MP 40 mg/day (0.83 mg/kg/day); etanercept before JAK	6 → 1 → 1	50% → 25% → 5%	24 h	96 h; 40→20 mg/day	8 days; stopped at discharge; no recurrence at 1 week; clinically diagnosed perioral herpes simplex
7	Tofacitinib 5 mg bid	Corticosteroids 96 h; MP 80 mg/day (1.23 mg/kg/day); no etanercept or IVIG	8 → 2 → 1	40% → 25% → 10%	48 h	72 h; 80→60 mg/day	28 days; stopped after outpatient continuation; no recurrence; corticosteroid-associated hyperglycemia requiring insulin
8	Upadacitinib 15 mg bid, then 15 mg qd	Corticosteroids 96 h; MP 60 mg/day (0.80 mg/kg/day); etanercept and IVIG before JAK	6 → 1 → 0	60% → 30% → 10%	New-lesion formation had ceased before JAK	96 h; 60→40 mg/day	61 days; stopped during corticosteroid taper; no recurrence, infection, or thrombosis during available follow-up
9	Baricitinib 2 mg tid, subsequently tapered	Corticosteroids 0 h; MP 40 mg/day (0.67 mg/kg/day); IVIG initiated concurrently	4 → 1 → 0	60% → 35% → 10%	72 h; transient day-5 flare, stabilization approximately day 9	312 h/13 d; 40→30 mg/day	Baricitinib March 29 to August 29; subsequently changed to upadacitinib after mild recurrence; hyperglycemia likely corticosteroid related; no new infection, thrombosis, or JAK-attributed hepatic/renal worsening

1. Cases 1–7 constituted the rescue treatment subgroup. All had continued new-lesion formation and at least one additional feature of active progression during the 24 h before JAK initiation despite at least 48 h of systemic corticosteroid treatment.

2. Case 8 was excluded from the time-to-cessation analysis because new-lesion formation had ceased before JAK initiation.

3. Case 9 was excluded from the rescue treatment subgroup because corticosteroids, IVIG, and baricitinib were initiated during the same treatment escalation phase.

4. “First documented absence of new lesions” refers to the first routine clinical assessment recording no further lesion formation and not the exact biological cessation time.

5. JAK regimens were individualized and off label and should not be interpreted as dosing recommendations.

6. The absence of a documented adverse event should not be interpreted as confirmation of safety when follow-up or laboratory monitoring was incomplete.

7. MP denotes methylprednisolone. The methylprednisolone dose at JAK inhibitor initiation is reported as mg/day (mg/kg/day), calculated from the recorded admission weight and rounded to two decimal places. Doses in the first reduction column also refer to methylprednisolone.

AE, adverse event; bid, twice daily; BSA, body surface area; HSV, herpes simplex virus; IVIG, intravenous immunoglobulin; JAK, Janus kinase; MP, methylprednisolone; qd, once daily; tid, three times daily; WI-NRS, Worst Itch Numerical Rating Scale.

*The 90% baseline BSA value in Case 4 predominantly reflected preexisting erythrodermic AD.

### 3.2 Early disease control in the rescue treatment subgroup

Cases 1–7 had received systemic corticosteroids for a median of 72 h before JAK inhibitor initiation (IQR, 48–96 h; range, 48–96 h). During the 24 h immediately preceding JAK initiation, all seven patients had documented continued new-lesion formation together with expanding BSA involvement, worsening erythema or edema, and uncontrolled severe pruritus. Five of seven patients had additionally received etanercept or IVIG before or at JAK inhibitor initiation.

After addition of an oral JAK inhibitor, the first documented absence of new lesions occurred within 24 h in 6 of 7 patients (85.7%; exact 95% CI, 42.1%–99.6%) and within 48 h in all 7 patients (100%; exact 95% CI, 59.0%–100%). The median time to the first documented absence of new lesions was 24 h (range, 24–48 h).

The first systemic corticosteroid dose reduction occurred at a median of 48 h after JAK inhibitor initiation (IQR, 24–72 h; range, 24–96 h). All seven patients began corticosteroid reduction within 96 h (100%; exact 95% CI, 59.0%–100%). Patient-level intervals between JAK initiation, first documented absence of new lesions, and first corticosteroid reduction are presented in Figure 1A, and aggregate rescue subgroup outcomes are summarized in Figure 1B and Table 3.

**Figure 1:**
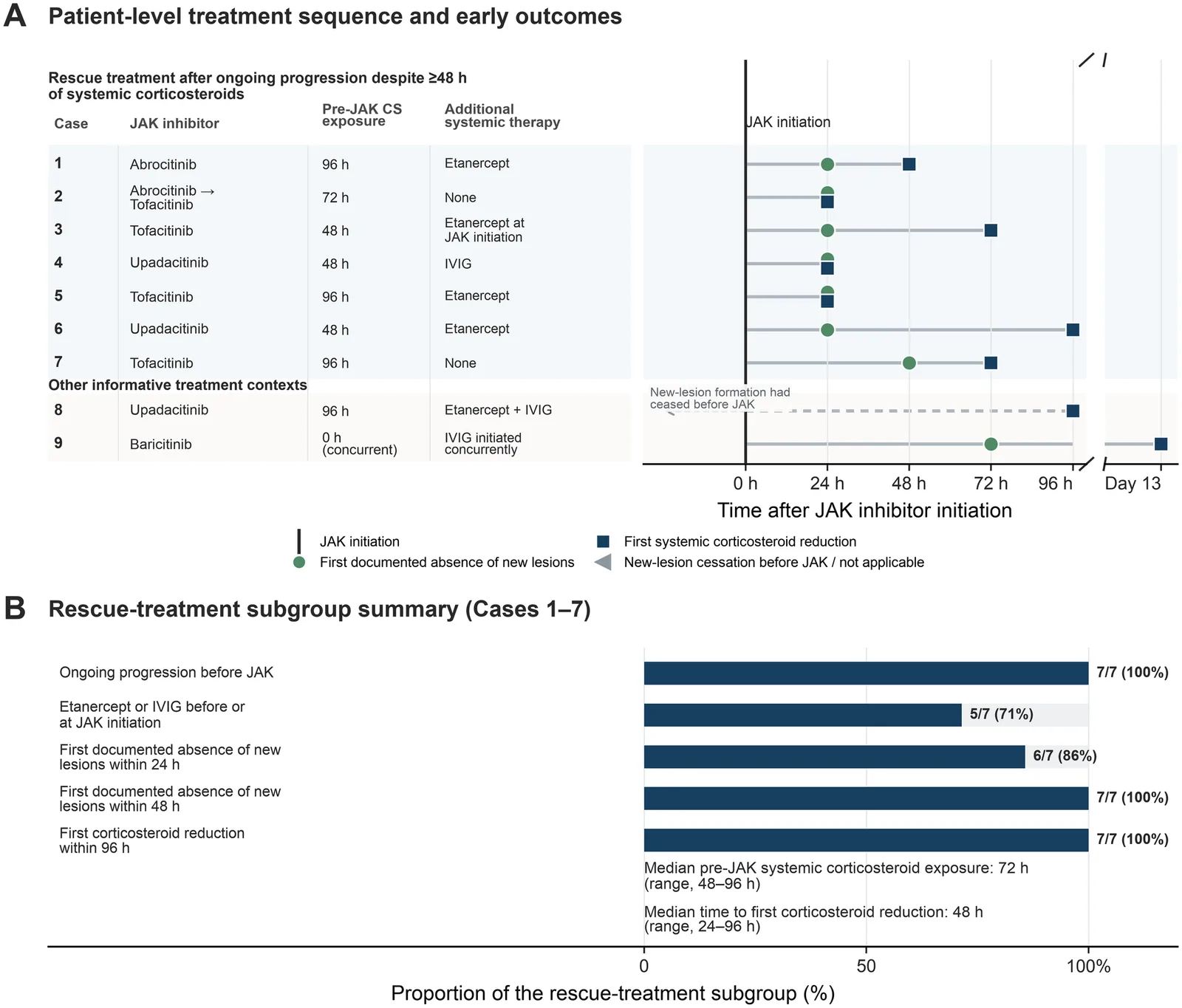
Patient-level treatment sequence and early disease control outcomes after oral Janus kinase inhibitor initiation. **(A)** Cases 1−7 comprised the rescue treatment subgroup and had documented ongoing disease progression despite 48−96 h of systemic corticosteroid treatment before JAK inhibitor initiation. Markers indicate the first documented absence of new lesions and the first reduction in systemic corticosteroid dose. Case 8 is displayed separately because new-lesion formation had ceased before JAK inhibitor initiation, although extensive cutaneous involvement and clinically important symptoms persisted. Case 9 is displayed separately because baricitinib, systemic corticosteroids, and intravenous immunoglobulin were initiated during the same treatment escalation phase; a transient flare occurred on day 5 before stabilization at approximately day 9. **(B)** Summary of the rescue treatment subgroup. The first documented absence of new lesions occurred within 24 h in six of seven patients (85.7%; exact 95% CI, 42.1%–99.6%) and within 48 h in all seven patients (100%; exact 95% CI, 59.0%–100%). All seven patients began systemic corticosteroid dose reduction within 96 h. Median systemic corticosteroid exposure before JAK inhibitor initiation was 72 h (range, 48−96 h), and the median interval from JAK inhibitor initiation to the first corticosteroid reduction was 48 h (range, 24−96 h). “First documented absence” refers to the first routine clinical assessment recording no new-lesion formation and does not represent the exact biological time at which lesion formation ceased. CS, corticosteroid; IVIG, intravenous immunoglobulin; JAK, Janus kinase.

**Table 3:** Aggregate early clinical outcomes after JAK inhibitor initiation.

A. Serial changes in the nine hospitalized patients							
Outcome	Baseline	24 h	48 h	72 h	Day 3	Day 7/discharge	Overall within-patient change
WI-NRS, *n* = 9	6 (5–7)	4 (3–5)	2 (2–3)	1 (1–2)	—	1 (0–1)	Friedman *χ*^2^ (4) = 35.04; *P* < 0.001; Kendall's *W* = 0.973
BSA involvement, %, *n* = 9	55 (50–60)	—	—	—	30 (25–30)	10 (5–10)	Friedman χ^2^ (2) = 18.00; *P* < 0.001; Kendall's *W* = 1.000
BSA sensitivity analysis excluding Case 4, %, *n* = 8	55 (47.5–60)	—	—	—	27.5 (23.75–30)	10 (5–10)	Friedman χ^2^ (2) = 16.00; *P* < 0.001; Kendall's *W* = 1.000

B. Early outcomes in the rescue treatment subgroup, Cases 1–7		
Outcome	Result	Exact 95% CI or range
Pre-JAK systemic corticosteroid exposure	72 h, median	IQR 48–96 h; range 48–96 h
First documented absence of new lesions within 24 h	6/7 (85.7%)	42.1%–99.6%
First documented absence of new lesions within 48 h	7/7 (100%)	59.0%–100%
Time to first documented absence of new lesions	24 h, median	Range 24–48 h
Time to first systemic corticosteroid reduction	48 h, median	IQR 24–72 h; range 24–96 h
Systemic corticosteroid reduction within 96 h	7/7 (100%)	59.0%–100%

Values in section A are median (interquartile range). Overall changes in WI-NRS and BSA were assessed using the Friedman test. After a significant overall test, baseline was compared with each follow-up time point using two-sided Wilcoxon signed-rank tests with exact *P* values from exhaustive sign permutations and Holm correction. All four WI-NRS comparisons with baseline remained significant after correction (adjusted *P* = 0.0156). Baseline-to-day 3 and baseline-to-day 7/discharge BSA comparisons also remained significant (adjusted *P* = 0.0078 for both comparisons). Exact confidence intervals for proportions were calculated using the Clopper–Pearson method.

The *P* values reflect within-patient temporal changes in the observed cohort and do not establish an independent JAK inhibitor treatment effect because there was no comparator group and most patients received concomitant systemic therapy.

BSA, body surface area; CI, confidence interval; IQR, interquartile range; JAK, Janus kinase; WI-NRS, Worst Itch Numerical Rating Scale.

### 3.3 Serial changes in pruritus severity

Complete serial WI-NRS assessments were available for all nine hospitalized patients. Median WI-NRS decreased from 6 (IQR, 5–7) at JAK inhibitor initiation to 4 (IQR, 3–5) at 24 h, 2 (IQR, 2–3) at 48 h, 1 (IQR, 1–2) at 72 h, and 1 (IQR, 0–1) by day 7 or discharge.

The overall within-patient change was significant [Friedman *χ*^2^(4) = 35.04, *P* < 0.001; Kendall's *W* = 0.973]. Comparisons of baseline with each follow-up time point remained significant after Holm correction, with an adjusted *P* = 0.0156 for each comparison.

The median reduction in WI-NRS at 72 h was 5 points (IQR, 4–5; range, 3–6), and all 9 patients achieved a reduction of at least 3 points by 72 h. Individual trajectories and the cohort median are shown in Figure 2A, and aggregate values are summarized in Table 3.

**Figure 2:**
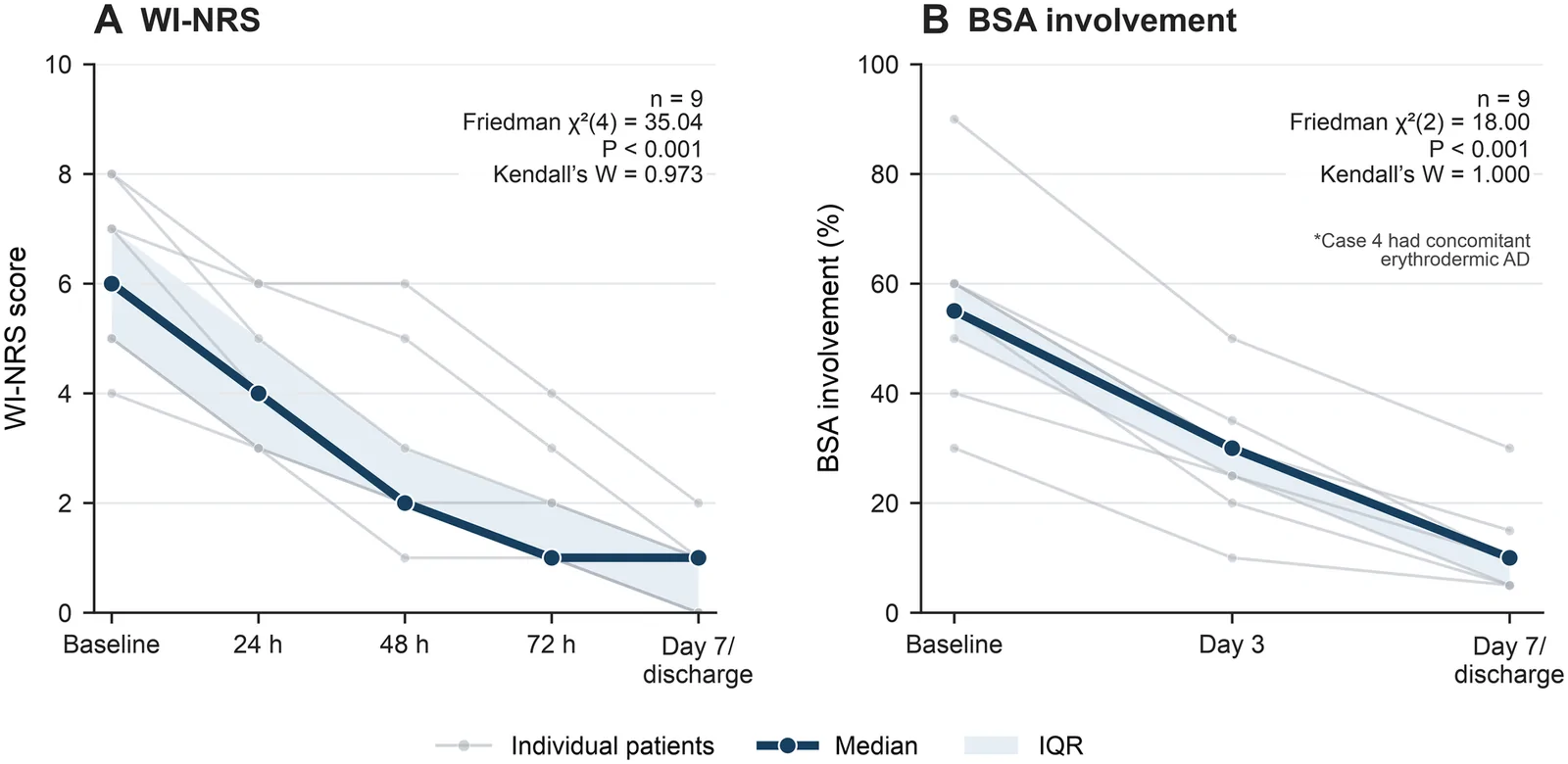
Serial changes in pruritus severity and cutaneous involvement during JAK inhibitor-containing treatment. **(A)** Individual Worst Itch Numerical Rating Scale (WI-NRS) trajectories in the nine hospitalized patients from baseline to 24, 48, and 72 h and to day 7 or discharge. Light gray lines represent individual patients, the dark line represents the cohort median, and the shaded area represents the interquartile range (IQR). WI-NRS decreased significantly over time [Friedman *χ*^2^(4) = 35.04, *P* < 0.001; Kendall's *W* = 0.973], and all nine patients achieved a reduction of at least three points by 72 h. **(B)** Individual trajectories of body surface area (BSA) involvement from baseline to day 3 and day 7 or discharge, displayed using the same format. BSA involvement decreased significantly over time [Friedman *χ*^2^(2) = 18.00, *P* < 0.001; Kendall's *W* = 1.000]. Because the baseline BSA in Case 4 predominantly reflected preexisting erythrodermic atopic dermatitis, a sensitivity analysis excluding this patient was performed and remained significant [Friedman *χ*^2^(2) = 16.00, *P* < 0.001]. Day 7/discharge denotes the day-7 assessment or the final inpatient assessment before discharge, whichever occurred first. These within-patient temporal changes do not establish an independent treatment effect of JAK inhibition.

### 3.4 Serial changes in cutaneous involvement

Median BSA involvement decreased from 55% (IQR, 50%–60%) at baseline to 30% (IQR, 25%–30%) at day 3 and 10% (IQR, 5%–10%) by day 7 or discharge. The median absolute reduction from baseline was 25 percentage points at day 3 (IQR, 25–30) and 50 percentage points by day 7 or discharge (IQR, 40–50).

The overall within-patient change was significant [Friedman *χ*^2^(2) = 18.00, *P* < 0.001; Kendall's *W* = 1.000]. Baseline-to-day 3 and baseline-to-day 7 or discharge comparisons remained significant after Holm correction, with adjusted *P* = 0.0078 for both comparisons.

In the sensitivity analysis excluding Case 4, median BSA involvement decreased from 55% (IQR, 47.5%–60%) at baseline to 27.5% (IQR, 23.75%–30%) at day 3 and 10% (IQR, 5%–10%) by day 7 or discharge. The overall change remained significant [Friedman *χ*^2^(2) = 16.00, *P* < 0.001; Kendall's *W* = 1.000]. Individual BSA trajectories are shown in Figure 2B. Overall changes in WI-NRS and BSA remained significant in within-patient permutation analyses, including the BSA analysis excluding Case 4 (all *P* < 0.001).

### 3.5 Other informative treatment contexts

Case 8 had definite DRESS with approximately 60% BSA involvement. New-lesion formation had ceased before upadacitinib initiation, but extensive erythema, desquamation, pruritus, and substantial disease burden persisted after treatment with methylprednisolone, etanercept, and IVIG. Upadacitinib was initiated as part of further treatment escalation and to facilitate corticosteroid reduction. WI-NRS decreased from 6 at baseline to 1 at 72 h and 0 by day 7 or discharge, while BSA involvement decreased from 60% to 30% at day 3 and 10% by day 7. The first corticosteroid reduction occurred 96 h after JAK inhibitor initiation.

Case 9 had definite voriconazole-associated DRESS with hepatic and renal involvement. Baricitinib, methylprednisolone, and IVIG were initiated during the same treatment escalation phase. New lesions remained documented at 24 h, status was not explicitly recorded at 48 h, and the first clearly documented absence of new lesions occurred at 72 h. A transient flare occurred on day 5, followed by stabilization at approximately day 9. WI-NRS decreased from 4 at baseline to 1 at 72 h and 0 by day 7 or discharge, while BSA involvement decreased from 60% to 35% at day 3 and 10% by day 7. The first corticosteroid reduction occurred 13 days after JAK inhibitor initiation. Because corticosteroids, IVIG, and baricitinib were initiated concurrently, the independent contribution of baricitinib could not be isolated.

### 3.6 Representative clinical changes, recurrence, and safety

Representative clinical photographs from Cases 2 and 8 demonstrated improvement in erythema, edema, desquamation, and lesional extent during JAK inhibitor-containing treatment (Figure 3). These images represent two distinct treatment contexts: rescue treatment after continued progression despite systemic corticosteroids in Case 2 and residual high disease burden with steroid-sparing escalation in Case 8. Additional clinical photographs of Case 9 at baseline and Day 10 are provided in Supplementary Figure S1.

**Figure 3:**
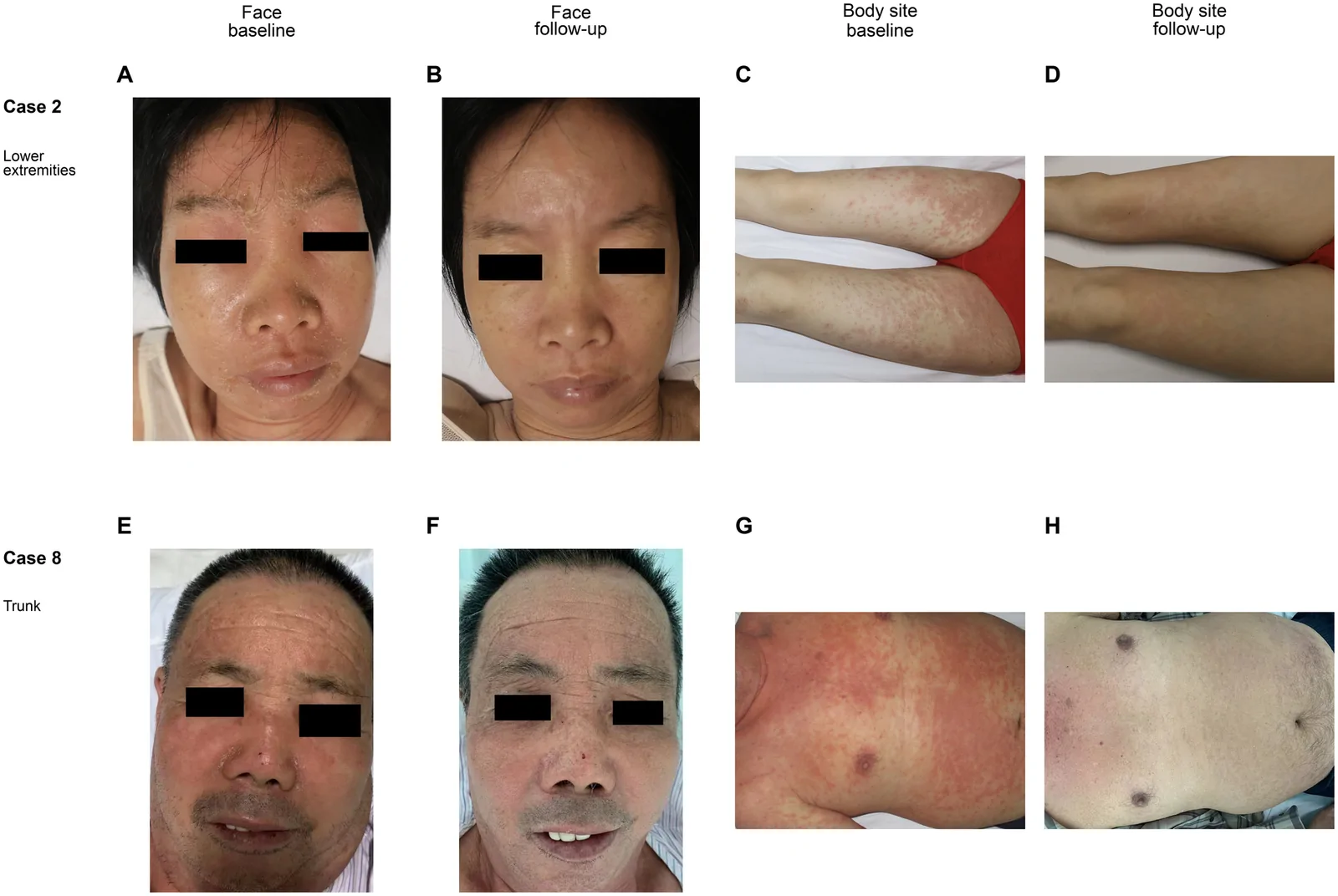
Representative clinical changes in selected treatment contexts. **(A–D)** Case 2, a patient in the rescue treatment subgroup, showing facial involvement at baseline **(A)** and follow-up **(B)**, and lower-extremity involvement at baseline **(C)** and follow-up **(D)**. **(E–H)** Case 8, who received JAK inhibition for residual high disease burden with steroid-sparing escalation, showing facial involvement at baseline **(E)** and follow-up **(F)**, and truncal involvement at baseline **(G)** and follow-up **(H)**. The follow-up images demonstrate improvement in erythema, edema, desquamation, and lesional extent during JAK inhibitor-containing treatment. Because systemic corticosteroids and other concomitant treatments were also administered, these clinical changes should not be interpreted as evidence of an independent JAK inhibitor effect. Written informed consent for publication of identifiable clinical photographs was obtained from both patients. JAK, Janus kinase.

No clear recurrence was documented in most patients during the available follow-up. Case 9 experienced the transient early flare described earlier and later developed mild recurrent erythema and pruritus during prolonged baricitinib treatment; these symptoms improved after switching to upadacitinib.

Two herpes simplex-related events were documented. Case 4 experienced recurrent HSV-2 in the setting of preexisting infection and extensive concomitant immunosuppression, and Case 6 developed clinically diagnosed perioral herpes simplex during combined upadacitinib and corticosteroid treatment. Cases 7 and 9 developed marked hyperglycemia that was considered primarily related to systemic corticosteroid treatment.

No new thrombosis, major cardiovascular event, or clear JAK-attributed hepatic or renal deterioration was documented. However, laboratory monitoring and follow-up duration were incomplete in several patients, and the absence of documented events should not be interpreted as evidence of safety.

## 4 Discussion

Progressive drug eruptions that continue to worsen despite culprit drug withdrawal and systemic corticosteroid treatment represent an important therapeutic challenge. In the present study, seven patients had persistent new-lesion formation, expanding cutaneous involvement, worsening erythema or edema, and severe pruritus after 48–96 h of systemic corticosteroid treatment. Following the addition of an oral JAK inhibitor, new-lesion formation was first documented as absent within 24 h in 6 patients and within 48 h in all 7. Systemic corticosteroid reduction was initiated within 96 h in every patient, with a median interval of 48 h. This consistent temporal pattern supports oral JAK inhibition as a potentially useful rescue strategy for selected patients with actively progressive drug eruptions.

The serial quantitative findings were concordant with the treatment sequence analysis. Across the 9 hospitalized patients, median WI-NRS decreased from 6 at baseline to 4 at 24 h, 2 at 48 h, and 1 at 72 h, and all patients achieved a reduction of at least 3 points by 72 h. The high Kendall's *W* value indicated a highly consistent direction of improvement across individual patients. Median BSA involvement decreased from 55% at baseline to 30% at day 3 and 10% by day 7 or discharge. The BSA result remained significant after exclusion of Case 4, whose baseline involvement was substantially influenced by preexisting erythrodermic atopic dermatitis. Together, the WI-NRS and BSA trajectories demonstrate that improvement was not only limited to the binary cessation of new lesions but also involved rapid relief of pruritus and progressive reduction in overall cutaneous disease burden.

The mechanistic rationale for JAK inhibition is particularly strong in DRESS/DIHS. Drug-responsive T cells may activate overlapping type 1, type 2, cytotoxic, memory, and pruritic inflammatory programs, many of which converge on the JAK-STAT pathway. Single-cell transcriptomic analysis of corticosteroid-refractory DIHS/DRESS demonstrated JAK-STAT activation in skin and circulating immune cells and guided successful treatment with tofacitinib (7). Recalcitrant DRESS has also been associated with CCR8/CCL1-mediated type 2 memory responses and CXCR3/CXCL10-mediated cytotoxic memory T-cell activation. Both inflammatory axes were attenuated by JAK inhibition in functional experiments (8). These findings provide a biological framework for the rapid improvement in pruritus, erythema, edema, and lesion formation observed in our patients.

Clinical evidence is also beginning to support this pathway-based approach. Tofacitinib has been reported to control severe DRESS with myocardial involvement (10). More recently, a six-patient series of recurrent or refractory DRESS treated with baricitinib or abrocitinib described improvement in cutaneous lesions and pruritus, reductions of more than 3 WI-NRS points, and initiation of corticosteroid tapering within 7–12 days (11). Our study extends these observations by focusing on very early response kinetics after treatment escalation. The clustering of new-lesion cessation within 24–48 h and the reduction in WI-NRS during the first 72 h suggest that the potential value of JAK inhibition may include rapid control of the active inflammatory phase, rather than only longer-term suppression of recurrent disease.

Cases 8 and 9 illustrate two additional roles for JAK inhibition within DRESS. In Case 8, new-lesion formation had already ceased, but extensive erythema, desquamation, pruritus, and substantial corticosteroid exposure persisted after methylprednisolone, etanercept, and IVIG. Further clinical improvement after upadacitinib and corticosteroid reduction 96 h later supports a potential role in controlling residual inflammatory burden and facilitating steroid tapering. In Case 9, baricitinib was initiated together with methylprednisolone and IVIG for severe DRESS with hepatic and renal involvement. The first documented absence of new lesions occurred at 72 h, followed by a transient flare on day 5 and stabilization at approximately day 9. This slower course suggests that response kinetics may vary with visceral involvement, baseline inflammatory burden, and disease stage at the time of treatment escalation.

Five of the seven rescue subgroup patients had also received etanercept or IVIG before or at JAK inhibitor initiation. Evidence for etanercept in severe cutaneous adverse reactions is strongest in SJS/TEN, whereas its role in DRESS, AGEP, and extensive maculopapular eruptions remains less well defined (6). In our patients, active progression remained documented when JAK inhibition was introduced. The subsequent improvement may reflect complementary suppression of multiple inflammatory pathways: upstream cytokine or cellular effects from preceding treatment together with broader downstream inhibition through JAK-STAT signaling. This possibility is clinically relevant because severe drug eruptions may involve simultaneous activation of type 1, type 2, cytotoxic, and pruritic pathways that are unlikely to be fully controlled by targeting a single mediator.

Evidence outside DRESS remains limited but biologically plausible. Persistent erythema multiforme has been associated with interferon-gamma- and interleukin-15-related inflammation, and improvement after tofacitinib or upadacitinib has been reported (12). This supports the use of JAK inhibition in Case 7, although the acute drug-associated EM-like phenotype differs from persistent erythema multiforme. Direct evidence for JAK inhibition in AGEP remains sparse and is less developed than in DRESS. AGEP is characterized by neutrophilic inflammation and may improve rapidly after culprit-drug withdrawal and systemic treatment (3). In Case 6, upadacitinib was added because the eruption remained extensive and progressive during systemic treatment, rather than on the basis of established AGEP-specific evidence. This response provides an exploratory clinical observation in a selected patient requiring treatment escalation. Upadacitinib-associated AGEP has also been reported (13), highlighting the need to reassess a new or worsening pustular eruption during treatment. Spatial proteomic analysis of TEN has also identified interferon-STAT1 signaling as a major pathogenic program and demonstrated therapeutic effects of JAK inhibition in experimental and clinical settings (9). Although none of our patients had SJS/TEN, these studies collectively suggest that JAK-STAT signaling may represent a shared downstream inflammatory node across several severe or persistent cutaneous drug reactions.

The present findings do not support routine systemic JAK inhibitor treatment for uncomplicated maculopapular eruptions, which generally improve after withdrawal of the culprit medication and symptomatic care. The non-DRESS patients in this cohort were selected because of extensive BSA involvement, severe pruritus, edema, continued progression during systemic treatment, or concern regarding prolonged corticosteroid exposure. In this subgroup, JAK inhibitors were added in response to continued progression during corticosteroid treatment, rather than on the basis of the drug eruption phenotype alone. The subsequent disease control and early corticosteroid reduction suggest a potential adjunctive benefit in this setting. These clinical features may help define a population in whom rapid pathway-directed treatment is most relevant. Future escalation criteria could incorporate persistent new-lesion formation, increasing BSA, severe itch, facial edema, systemic abnormalities, and failure to improve after a predefined period of corticosteroid treatment.

The early corticosteroid reduction observed in the rescue subgroup represents another clinically important finding. Prolonged systemic corticosteroid treatment may be accompanied by infection, hyperglycemia, hypertension, osteoporosis, and relapse during tapering. In our cohort, all seven rescue subgroup patients began corticosteroid reduction within 96 h after JAK inhibitor initiation. The median interval of 48 h was shorter than the 7–12 days reported before initial tapering in the published six-patient DRESS series (11), although differences in phenotype, disease severity, treatment sequence, tapering practice, and outcome definition should be considered. Selected clinical studies are summarized alongside the present series in Supplementary Table S1, with the disease phenotype, corticosteroid regimen, concomitant treatment, and reported response and tapering endpoints presented for clinical context. The present results support the inclusion of corticosteroid-related endpoints in future studies, including time to first reduction, cumulative prednisone-equivalent exposure, percentage dose reduction, time to discontinuation, and relapse during tapering.

The potential rescue and steroid-sparing roles of JAK inhibition should also be viewed in the context of other systemic treatment options beyond corticosteroids. Ciclosporin, for example, can be considered for corticosteroid-refractory DRESS (2). Retrospective studies have reported rapid disease control with ciclosporin; a comparison of 27 ciclosporin-treated and 53 corticosteroid-treated patients found no significant differences in hospitalization, treatment duration, or time to clinical normalization (14, 15). JAK inhibition offers a different pharmacologic approach by targeting cytokine-receptor signaling rather than calcineurin-mediated T-cell activation (7, 8, 14). Clinical responses have also been reported in prolonged DRESS after inadequate control with other immunosuppressants, including ciclosporin (8). In our rescue subgroup, the rapid disease control and early corticosteroid reduction observed after JAK initiation support its potential role as an additional treatment option. Direct comparisons are needed to determine its relative efficacy and safety versus ciclosporin.

Four oral JAK inhibitors were used in this series. Tofacitinib preferentially inhibits JAK1/JAK3, baricitinib predominantly inhibits JAK1/JAK2, and upadacitinib and abrocitinib are relatively JAK1-preferential, although selectivity varies with dose and systemic exposure (16, 17). The similar direction of improvement across these agents supports a pathway-level therapeutic signal. The use of different agents reflected individualized clinical selection within this treatment approach; the study focused on adjunctive JAK inhibition for disease control and corticosteroid reduction rather than comparison among individual agents. At the same time, differences in selectivity, pharmacokinetics, comorbidities, organ involvement, infection risk, and treatment duration may influence agent selection. Larger studies will be required to determine whether particular phenotypes or inflammatory signatures respond preferentially to specific JAK inhibitors.

Safety remains important when JAK inhibitors are combined with systemic corticosteroids and other immunomodulatory treatments. Two herpes simplex-related events were documented. Case 4 experienced recurrent HSV-2 in the setting of preexisting infection and extensive concomitant immunosuppression, whereas Case 6 developed clinically diagnosed perioral herpes simplex during combined upadacitinib and corticosteroid treatment. Oral JAK inhibitors are associated with an increased risk of infection, including herpesvirus infection in dermatologic populations (18). Two patients developed marked hyperglycemia that was considered primarily corticosteroid related. No new thrombosis, major cardiovascular event, or clear JAK-attributed hepatic or renal deterioration was documented. For selected patients, baseline evaluation should include screening for active infection and assessment of hematologic, hepatic, renal, cardiovascular, thrombotic, and malignancy risk, followed by appropriate clinical and laboratory monitoring (19, 20).

This study has several strengths. It reconstructed the treatment sequence before and after JAK inhibitor initiation rather than simply reporting improvement following exposure. A clinically coherent rescue treatment subgroup was separated from other treatment contexts, avoiding inappropriate pooling of patients who received JAK inhibition for different indications. Serial WI-NRS and BSA data enabled formal within-patient repeated-measures analyses, while the sensitivity analysis excluding Case 4 addressed an important source of confounding. The timing of new-lesion cessation and corticosteroid reduction was evaluated at the patient level, and the clinical data were independently verified by two dermatologists. Representative photographs provided additional support for the observed improvement.

Several limitations should be acknowledged. This was a small, single-center, retrospective, and uncontrolled study with heterogeneous clinical phenotypes, individualized JAK regimens, and substantial concomitant treatment. The rescue treatment definition and additional treatment-context categories were assigned retrospectively and require prospective validation. Culprit drug withdrawal, systemic corticosteroids, etanercept, IVIG, supportive treatment, regression to the mean, and the natural course of the eruptions may all have contributed to improvement. New-lesion cessation was based on intermittent clinical assessments, and WI-NRS and BSA values were retrospectively reconstructed from routine records rather than prospectively collected using a standardized protocol. Diagnostic certainty and culprit attribution varied, particularly in the DRESS-like, EM-like, probable AGEP, and erythrodermic AD-associated cases. Follow-up and laboratory monitoring were incomplete, limiting assessment of recurrence and uncommon or delayed adverse events. The sample size also precluded comparison among JAK inhibitors or determination of optimal dosing and treatment duration.

Prospective studies should stratify patients by phenotype and treatment context, define objective criteria for treatment escalation, standardize concomitant corticosteroid treatment and tapering, and collect new-lesion status, WI-NRS, BSA, disease-specific severity scores, and photographs at predefined early time points. Controlled or matched-cohort designs will be necessary to quantify the incremental benefit of JAK inhibition. Incorporating immune biomarkers—including interleukin-5, CCL1, CXCL10, interferon-associated proteins, phosphorylated STAT1, drug-reactive T-cell populations, and herpesvirus reactivation—may help identify patients most likely to benefit and clarify whether different drug-eruption phenotypes share a treatment-responsive JAK-STAT signature.

## 5 Conclusion

In this retrospective observational case series, adjunctive treatment with oral JAK inhibitors was associated with rapid cessation of new-lesion formation, substantial improvement in pruritus and cutaneous involvement, and early corticosteroid reduction in hospitalized patients with extensive or difficult-to-control drug eruptions. Although causality cannot be established, these findings suggest that JAK inhibitors may represent a promising rescue or steroid-sparing therapeutic option for selected difficult-to-control drug eruptions and support further investigation in larger, controlled prospective studies.

## Data Availability

The datasets presented in this article are not readily available because the de-identified patient-level clinical data are subject to institutional and ethical restrictions due to the retrospective use of medical records and the potentially sensitive nature of the health information. Requests to access the datasets should be directed to PW at wangpeng02@126.com.

## References

[1] Duong TA Valeyrie-Allanore L Wolkenstein P Chosidow O. Severe cutaneous adverse reactions to drugs. Lancet. (2017) 390:1996–2011. 10.1016/S0140-6736(16)30378-6 28476287

[2] Brüggen M-C Walsh S Ameri MM Anasiewicz N Maverakis E French LE. Management of adult patients with drug reaction with eosinophilia and systemic symptoms: a delphi-based international consensus. JAMA Dermatol. (2024) 160:37–44. 10.1001/jamadermatol.2023.4450 PMC10652220 37966824

[3] Tetart F Walsh S Milpied B Gaspar K Vorobyev A Tiplica GS. Acute generalized exanthematous pustulosis: european expert consensus for diagnosis and management. J Eur Acad Dermatol Venereol. (2024) 38:2073–81. 10.1111/jdv.20232 39023187

[4] Wei BM Fox LP Kaffenberger BH Korman AM Micheletti RG Mostaghimi A. Drug-induced hypersensitivity syndrome/drug reaction with eosinophilia and systemic symptoms. Part II: Diagnosis and Management. J Am Acad Dermatol. (2024) 90:911–26. 10.1016/j.jaad.2023.02.073 37516356

[5] Wang S Kang Y He C Jin H. The systemic treatments for drug reaction with eosinophilia and systemic symptoms (DRESS) beyond corticosteroids. World Allergy Organ J. (2024) 17:100935. 10.1016/j.waojou.2024.100935 39156598 PMC11325795

[6] Wang C-W Yang L-Y Chen C-B Ho H-C Hung S-I Yang C-H. Randomized, controlled trial of TNF-α antagonist in CTL-mediated severe cutaneous adverse reactions. J Clin Invest. (2018) 128:985–96. 10.1172/JCI93349 29400697 PMC5824923

[7] Kim D Kobayashi T Voisin B Jo J-H Sakamoto K Jin S-P. Targeted therapy guided by single-cell transcriptomic analysis in drug-induced hypersensitivity syndrome: a case report. Nat Med. (2020) 26:236–43. 10.1038/s41591-019-0733-7 31959990 PMC7105105

[8] Chiu T-M Chen C-B Lu C-W Hui RC-Y Chi M-H Chang Y-C. CCR8/CCL1 and CXCR3/CXCL10 axis-mediated memory T-cell activation in patients with recalcitrant drug-induced hypersensitivity. Br J Dermatol. (2025) 192:293–305. 10.1093/bjd/ljae375 39503255

[9] Nordmann TM Anderton H Hasegawa A Schweizer L Zhang P Stadler P-C. Spatial proteomics identifies JAKi as treatment for a lethal skin disease. Nature. (2024) 635:1001–9. 10.1038/s41586-024-08061-0 39415009 PMC11602713

[10] Damsky WE Vesely MD Lee AI Choi J Meyer A-C Chen M. Drug-induced hypersensitivity syndrome with myocardial involvement treated with tofacitinib. JAAD Case Rep. (2019) 5:1018–26. 10.1016/j.jdcr.2019.07.004 31763425 PMC6864390

[11] Liu W Wang Y Wang J Xu H Shen X Zheng J. Janus kinase 1 and 1/2 inhibitors for treating drug reaction with eosinophilia and systemic symptoms: a case series. Clin Exp Dermatol. (2026) 51:1076–82. 10.1093/ced/llag072 41652871

[12] Murphy MJ Gruenstein D Wang A Peterson D Levitt J King B. Treatment of persistent erythema multiforme with janus kinase inhibition and the role of interferon gamma and interleukin 15 in its pathogenesis. JAMA Dermatol. (2021) 157:1477–82. 10.1001/jamadermatol.2021.4084 34757416 PMC8581795

[13] Banda T Butt S Maheshwari M Chattopadhyay M. Acute generalised exanthematous pustulosis associated with upadacitinib treatment. Skin Health Dis. (2024) 4:e444. 10.1002/ski2.444 39624768 PMC11608896

[14] Nguyen E Yanes D Imadojemu S Kroshinsky D. Evaluation of cyclosporine for the treatment of DRESS syndrome. JAMA Dermatol. (2020) 156:704–6. 10.1001/jamadermatol.2020.0048 32159726 PMC7066519

[15] Kwon HJ Yoon JH. Comparison of cyclosporine and systemic corticosteroid for treating drug reaction with eosinophilia and systemic symptoms syndrome: a retrospective 20-year single-centre study in South Korea. Australas J Dermatol. (2023) 64:50–7. 10.1111/ajd.13982 36695042

[16] Traves PG Murray B Campigotto F Galien R Meng A Di Paolo JA. JAK selectivity and the implications for clinical inhibition of pharmacodynamic cytokine signalling by filgotinib, upadacitinib, tofacitinib and baricitinib. Ann Rheum Dis. (2021) 80:865–75. 10.1136/annrheumdis-2020-219012. PMID: 33741556. 33741556 PMC8237188

[17] Mohamed MEF Beck D Camp HS Othman AA. Preferential inhibition of JAK1 relative to JAK3 by upadacitinib: exposure-response analyses of *ex vivo* data from 2 phase 1 clinical trials and comparison to tofacitinib. J Clin Pharmacol. (2020) 60:188–97. 10.1002/jcph.1513 31448433 PMC6973126

[18] Isufi D Jensen MB Loft N Skov L Elberling J Alinaghi F. Risk of infections during treatment with oral janus kinase inhibitors in randomized placebo-controlled trials: a systematic review and meta-analysis. JAAD Int. (2025) 18:106–16. 10.1016/j.jdin.2024.09.012 39717054 PMC11664075

[19] Ytterberg SR Bhatt DL Mikuls TR Koch GG Fleischmann R Rivas JL. Cardiovascular and cancer risk with tofacitinib in rheumatoid arthritis. N Engl J Med. (2022) 386:316–26. 10.1056/NEJMoa2109927 35081280

[20] Samuel C Cornman H Kambala A Kwatra SG. A review on the safety of using JAK inhibitors in dermatology: clinical and laboratory monitoring. Dermatol Ther (Heidelb). (2023) 13:729–49. 10.1007/s13555-023-00892-5 36790724 PMC9930707

