# Supplementary material for: Efficacy of adjunctive oral Janus kinase inhibitors in hospitalized patients with extensive or difficult-to-control drug eruptions: a retrospective observational case series
Source: Front Med (Lausanne). 2026 Oct 26;13:1947886. doi: 10.3389/fmed.2026.1947886 (PMC13745343; doi:10.3389/fmed.2026.1947886)
Supplement: Supplementary file 1 [file Image1.pdf]

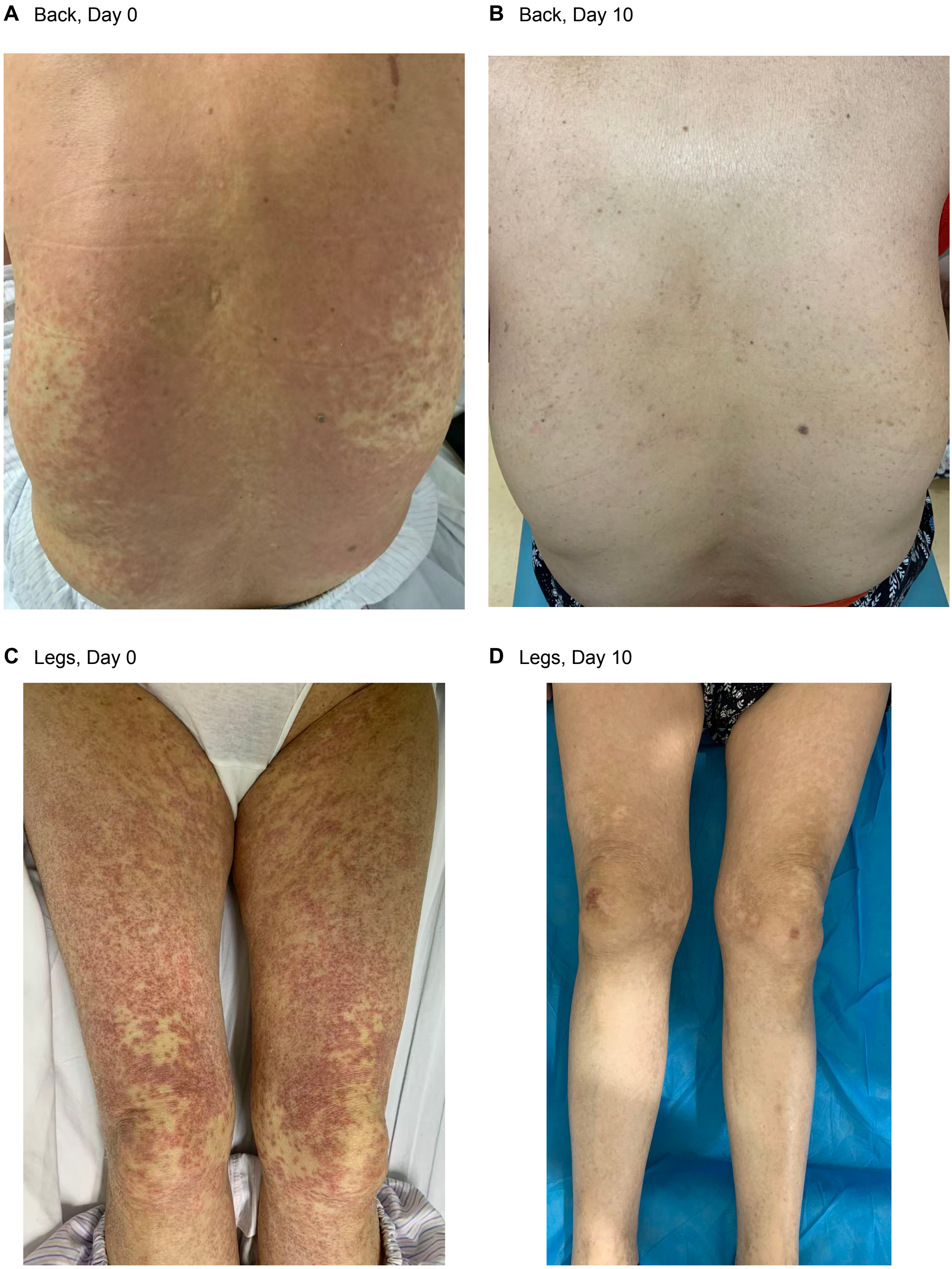


**Supplementary Figure S1. Additional clinical photographs of Case 9 at baseline and Day 10.**

Clinical photographs of the back (A, B) and lower extremities (C, D) on the day of JAK inhibitor initiation (Day 0; A, C) and on Day 10 (B, D), showing marked improvement in erythema and cutaneous lesions. Treatment included baricitinib, methylprednisolone, and intravenous immunoglobulin. Written informed consent for publication was obtained from the patient.
