# Supplementary material for: Efficacy of adjunctive oral Janus kinase inhibitors in hospitalized patients with extensive or difficult-to-control drug eruptions: a retrospective observational case series
Source: Front Med (Lausanne). 2026 Oct 26;13:1947886. doi: 10.3389/fmed.2026.1947886 (PMC13745343; doi:10.3389/fmed.2026.1947886)
Supplement: Supplementary file 2 [file Table1.docx]

**Supplementary Table S1**

Clinical context, corticosteroid regimens, and response outcomes in the present series and selected published studies of drug eruptions.

**A. Present series: distinct treatment contexts**

| **Study and population** | **Diagnostic assessment and disease burden** | **Treatment context and other therapy** | **Corticosteroid regimen and time point** | **Clinical response endpoint and timing** | **Corticosteroid reduction / withdrawal** |
| --- | --- | --- | --- | --- | --- |
| **Present series**  Rescue subgroup  Cases 1–7; n = 7 | MPE/morbilliform eruptions, EM-like eruption, probable AGEP, and suspected MPE on erythrodermic AD.  Baseline BSA: 30–90%; the 90% value mainly reflected pre-existing AD. | Abrocitinib, tofacitinib or upadacitinib added after 48–96 h of systemic CS with continuing new lesions.  Five of seven also received etanercept or IVIG before or at JAK initiation. | At JAK initiation:  MP 40–80 mg/day  (0.81–1.90 mg/kg/day).  Weight-adjusted doses use the recorded admission weights. | From JAK initiation to first documented absence of new lesions:  6/7 within 24 h;  7/7 within 48 h. | First reduction after JAK initiation: median 48 h (range, 24–96 h).  Complete withdrawal time was not systematically reported. |
| **Present series**  Case 8; n = 1 | Definite DRESS; RegiSCAR 7.  BSA: 60%.  No definite internal-organ involvement. | Upadacitinib added for residual disease burden and to facilitate CS reduction after MP, etanercept and IVIG.  New-lesion formation had already ceased. | At JAK initiation:  MP 60 mg/day  (0.80 mg/kg/day).  Pre-JAK CS exposure: 96 h. | New-lesion cessation after JAK: not applicable.  WI-NRS: 6 to 1 at 72 h.  BSA: 60% to 30% at day 3. | First reduction at 96 h after JAK initiation: MP 60 to 40 mg/day.  Complete withdrawal time not reported. |
| **Present series**  Case 9; n = 1 | Definite DRESS; RegiSCAR 7.  BSA: 60%.  Hepatic and renal involvement. | Baricitinib, MP and IVIG initiated during the same treatment-escalation phase.  Excluded from the rescue subgroup. | At JAK initiation:  MP 40 mg/day  (0.67 mg/kg/day).  Pre-JAK CS exposure: 0 h. | First documented absence of new lesions at 72 h; transient flare on day 5, followed by stabilization at approximately day 9. | First reduction at 13 days after JAK initiation: MP 40 to 30 mg/day.  Complete withdrawal time not reported. |

Body-weight-adjusted MP doses were calculated using recorded admission weights and rounded to two decimal places. Patient-level treatment sequences are provided in Table 2.

**Supplementary Table S1 (continued)**

**B. Published JAK-inhibitor studies in DRESS/DIHS**

| **Study and population** | **Diagnostic assessment and disease burden** | **Treatment context and other therapy** | **Corticosteroid regimen and time point** | **Clinical response endpoint and timing** | **Corticosteroid reduction / withdrawal** |
| --- | --- | --- | --- | --- | --- |
| **Liu et al. (2026) (1)**  n = 6; ages 9–70 years [a] | RegiSCAR 6–7.  Author-graded moderate DRESS (n = 5) or severe DRESS (n = 1).  All had liver involvement; two had haemophagocytic manifestations. | Baricitinib (n = 4) or abrocitinib (n = 2) added 15–70 days after CS initiation. All had flares during CS treatment/tapering.  Other treatments included IVIG; prior adalimumab in patients 1 and 6 and topical CS in patients 3 and 4. | Pre-JAK treatment: MP pulses of 20 then 10 mg/kg/day (patient 1); prednisone 0.5 mg/kg/day (patients 3 and 4).  Patient 6 received MP 60 mg/day at JAK addition.  A cohort-wide JAK-start dose in mg/kg/day was not reported. | Next-day itch improvement in patients 3 and 4; skin clearance by week 4 in patient 1.  WI-NRS decreased by >3 points in five evaluable patients.  No cohort-level new-lesion cessation time. | First successful taper: 7–12 days after JAK initiation.  Complete withdrawal: 4/6 within 28–62 days; 2/6 remained on low-dose CS for underlying autoimmune disease. |
| **Chiu et al. (2025) (2)**  n = 7 treated with JAK inhibitors | JAK-treated subset of a 32-patient prolonged-DRESS cohort.  Prolonged disease: treatment for ≥8 weeks.  RegiSCAR-based diagnosis. | Tofacitinib; one nonresponder switched to upadacitinib after 3 weeks.  Prior therapies included CS, ciclosporin or dupilumab.  No concurrent immunosuppressants during JAK treatment. | Prior weight-adjusted CS regimen for the seven JAK-treated patients was not separately reported in the main text. | Six recovered within 2 weeks of tofacitinib.  One achieved complete remission 60 days after starting upadacitinib. | First CS reduction after JAK initiation: not applicable; no concurrent CS was used. |
| **Damsky et al. (2019) (3)**  n = 2 adults; recurrent episodes | DIHS/DRESS with myocarditis; initial RegiSCAR scores 4 and 5.  Patient 1 also developed severe neurological involvement. | Tofacitinib with variable CS, ciclosporin or methotrexate; MP pulses/IVIG at flares.  Patient 2 had recurrent rash on prednisone plus methotrexate. | Patient 1: initial prednisone 1 mg/kg/day; 120 mg/day at the first tofacitinib addition (supplement).  Patient 2: prednisone 40 mg/day at addition.  Regimens varied across episodes. | No standardized new-lesion cessation endpoint.  Patient 1’s later relapse responded promptly to tofacitinib monotherapy. Both had recurrence after tofacitinib withdrawal and improvement after restarting. | Patient 2: prednisone and methotrexate withdrawn over 8 weeks after tofacitinib initiation.  Patient 1: protracted treatment with repeated flares; no single first-reduction interval. |

[a] Liu et al.: the CS pulse doses shown refer to initial treatment, not the JAK-start regimen. Abrocitinib dosing differs between the narrative and Table 1/Figure 3, so its dose is not reproduced here.

**Supplementary Table S1 (continued)**

**C. Conventional-treatment context in DRESS and AGEP**

| **Study and population** | **Diagnostic assessment and disease burden** | **Treatment context and other therapy** | **Corticosteroid regimen and time point** | **Clinical response endpoint and timing** | **Corticosteroid reduction / withdrawal** |
| --- | --- | --- | --- | --- | --- |
| **Nguyen et al. (2020) (4)**  Ciclosporin, n = 5; systemic CS, n = 21 | Adult DRESS; RegiSCAR >5.  All had BSA >50%.  Hepatic involvement: 4/5 versus 16/21. | Retrospective comparison of ciclosporin and systemic CS.  Selection included CS contraindications.  Mean disease-onset-to-treatment intervals: 6.5 versus 6 days. | CS arm: intravenous MP 1–2 mg/kg/day, followed by variable prednisone tapering. | From treatment initiation to halted progression: mean (SD) 2.0 (1.2) versus 2.8 (2.3) days.  Endpoint: cessation of eruption worsening. | First CS reduction: NR.  Treatment duration, mean (SD): ciclosporin 12.5 (4.0) days; CS 48.5 (53.0) days.  These are total treatment durations, not first-taper times. |
| **Thienvibul et al. (2015) (5)**  n = 19 [b] | EuroSCAR ≥5: 14 definite and 5 probable cases.  17 generalized and 2 localized eruptions; hepatitis in 5/19. | Culprit-drug withdrawal when applicable.  Topical CS, n = 11; oral prednisolone, n = 6; supportive care, n = 2.  16 inpatients and 3 outpatients. | Oral prednisolone dose and mg/kg/day were not reported. | From drug cessation to pustule resolution: median 3 days (range, 2–12).  Treatment-group medians: topical CS 2 days; oral prednisolone 3 days; supportive care 2.5 days. | Tapering duration: NR.  One patient with a prolonged erythematous/desquamative course required gradual systemic CS tapering. |

[b] Eighteen cases were drug-associated; one had a suspected viral trigger. The original report included two localized eruptions.

Interpretation: this is a selected descriptive overview, not a matched historical control. Time origins and endpoint definitions are retained as reported; first dose reduction, complete withdrawal and total treatment duration are distinct outcomes. CS drugs and dose time points are specified without cross-agent conversion. NR = not reported for the stated population or endpoint. RegiSCAR/EuroSCAR scores describe diagnostic assessment, not a common severity scale.

Abbreviations: AD, atopic dermatitis; AGEP, acute generalized exanthematous pustulosis; BSA, body surface area; CS, corticosteroid; DIHS, drug-induced hypersensitivity syndrome; DRESS, drug reaction with eosinophilia and systemic symptoms; EM, erythema multiforme; IVIG, intravenous immunoglobulin; JAK, Janus kinase; MP, methylprednisolone; MPE, maculopapular eruption; SD, standard deviation; WI-NRS, Worst Itch Numerical Rating Scale.

**References for Supplementary Table S1**

1. Liu W, Wang Y, Wang J, Xu H, Shen X, Zheng J, et al. Janus kinase 1 and 1/2 inhibitors for treating drug reaction with eosinophilia and systemic symptoms: a case series. Clin Exp Dermatol. (2026) 51:1076–1082. doi: 10.1093/ced/llag072

2. Chiu TM, Chen CB, Lu CW, Hui RC, Chi MH, Chang YC, et al. CCR8/CCL1 and CXCR3/CXCL10 axis-mediated memory T-cell activation in patients with recalcitrant drug-induced hypersensitivity. Br J Dermatol. (2025) 192:293–305. doi: 10.1093/bjd/ljae375

3. Damsky WE, Vesely MD, Lee AI, Choi J, Meyer AC, Chen M, et al. Drug-induced hypersensitivity syndrome with myocardial involvement treated with tofacitinib. JAAD Case Rep. (2019) 5:1018–1026. doi: 10.1016/j.jdcr.2019.07.004

4. Nguyen E, Yanes D, Imadojemu S, Kroshinsky D. Evaluation of cyclosporine for the treatment of DRESS syndrome. JAMA Dermatol. (2020) 156:704–706. doi: 10.1001/jamadermatol.2020.0048

5. Thienvibul C, Vachiramon V, Chanprapaph K. Five-year retrospective review of acute generalized exanthematous pustulosis. Dermatol Res Pract. (2015) 2015:260928. doi: 10.1155/2015/260928
